# Supplementary material for: Integration of ATAC-seq and RNA-seq Unravels Chromatin Accessibility during Sex Reversal in Orange-Spotted Grouper (Epinephelus coioides)
Source: Int J Mol Sci. 2020 Apr 17;21(8):2800. doi: 10.3390/ijms21082800 (PMC7215633; doi:10.3390/ijms21082800)
Supplement: Supplementary file 1 [file ijms-21-02800-s001.zip › ijms-747820 ▓╣│Σ▓─┴╧╖ó▒φ/Figure S1-S5.pdf]

Article

Integration of ATAC-seq and RNA-seq Unravels Chromatin Accessibility during Sex Reversal in Orange-Spotted Grouper (*Epinephelus coioides*)

Xi Wu [1], Yang Yang [1], Chaoyue Zhong [1], Yin Guo [1], Tengyu Wei [1], Shuisheng Li [1,*], Haoran Lin [1] and Xiaochun Liu [1,2,*]

[1] State Key Laboratory of Biocontrol, Guangdong Province Key Laboratory for Improved Variety Reproduction of Aquatic Economic Animals, Institute of Aquatic Economic Animals, School of Life Sciences, Sun Yat-Sen University, Guangzhou 510275, China; wuxi577@126.com (X.W.); yyang0103@163.com (Y.Y.); zhongchy9@mail2.sysu.edu.cn (C.Z.); guoyin10@163.com (Y.G.); weitengyu1996@163.com (T.W.); lsslhr@mail.sysu.edu.cn (H.L.)

[2] Southern Laboratory of Ocean Science and Engineering, Zhuhai 519000, China

* Correspondence: lshuish@mail.sysu.edu.cn (S.L.); lsslxc@mail.sysu.edu.cn (X.L.); Tel. +86-020-8411-2511(X.L.).

Received: 1 March 2020; Accepted: 15 April 2020; Published: April 2020

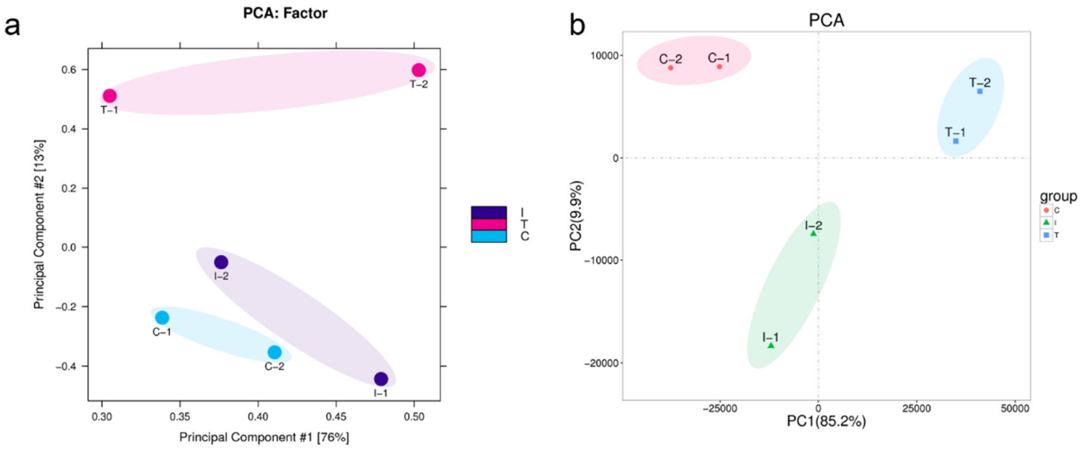

Figure S1. The PCA plot of the six samples in ATAC-seq (**a**) and RNA-seq (**b**).

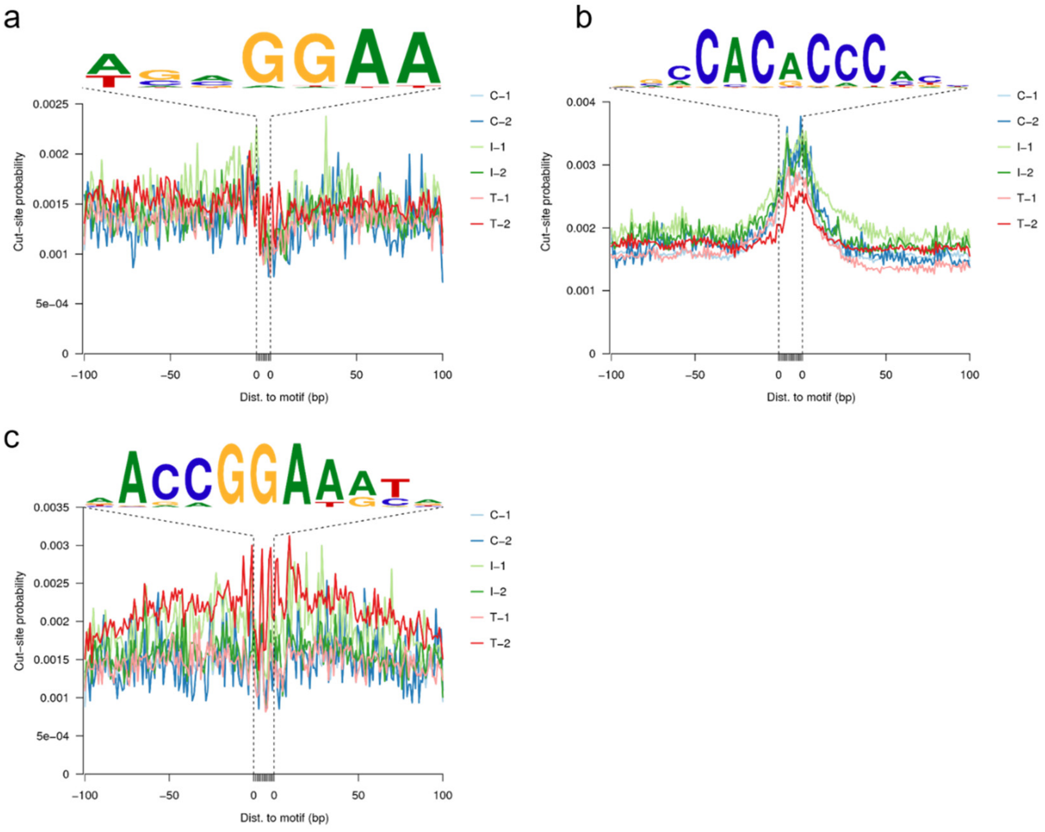

Figure S2. The footprints of SPIB **(a)**, KLF9 **(b)**, and ETV2 **(c)** in ATAC-seq data.

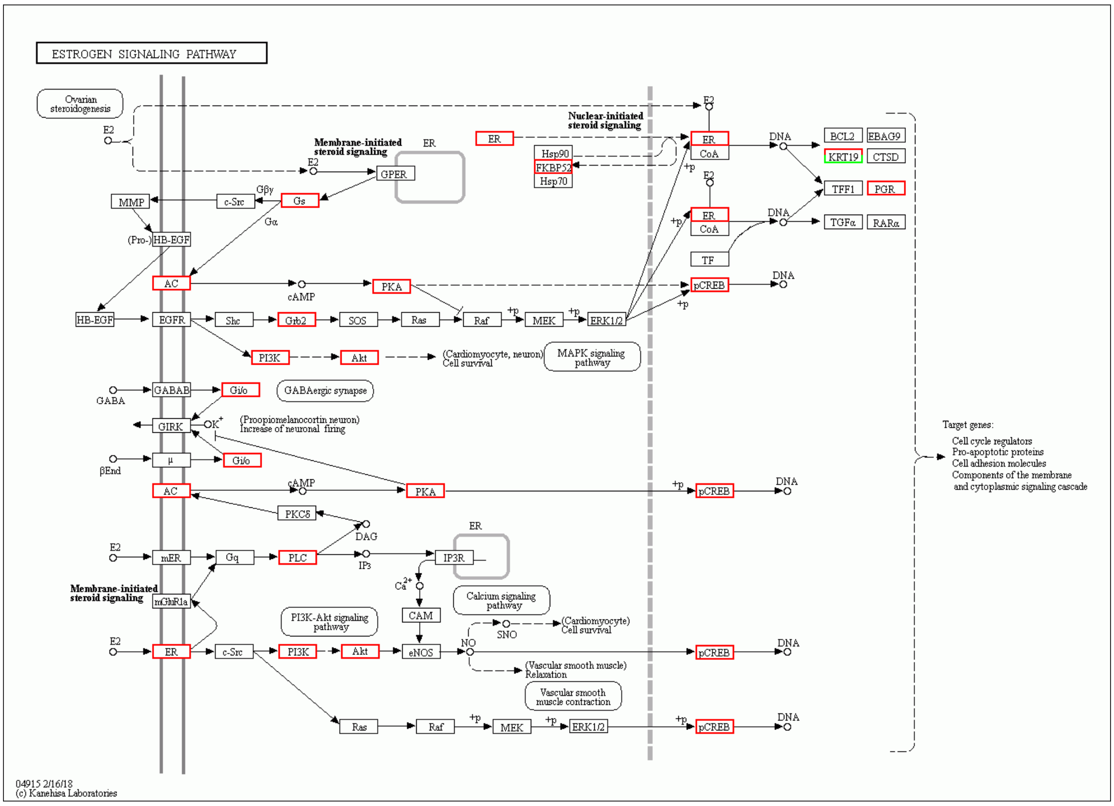

Figure S3. The map of estrogen signaling pathway in comparison of Control group and Intersex group in RNA-seq.

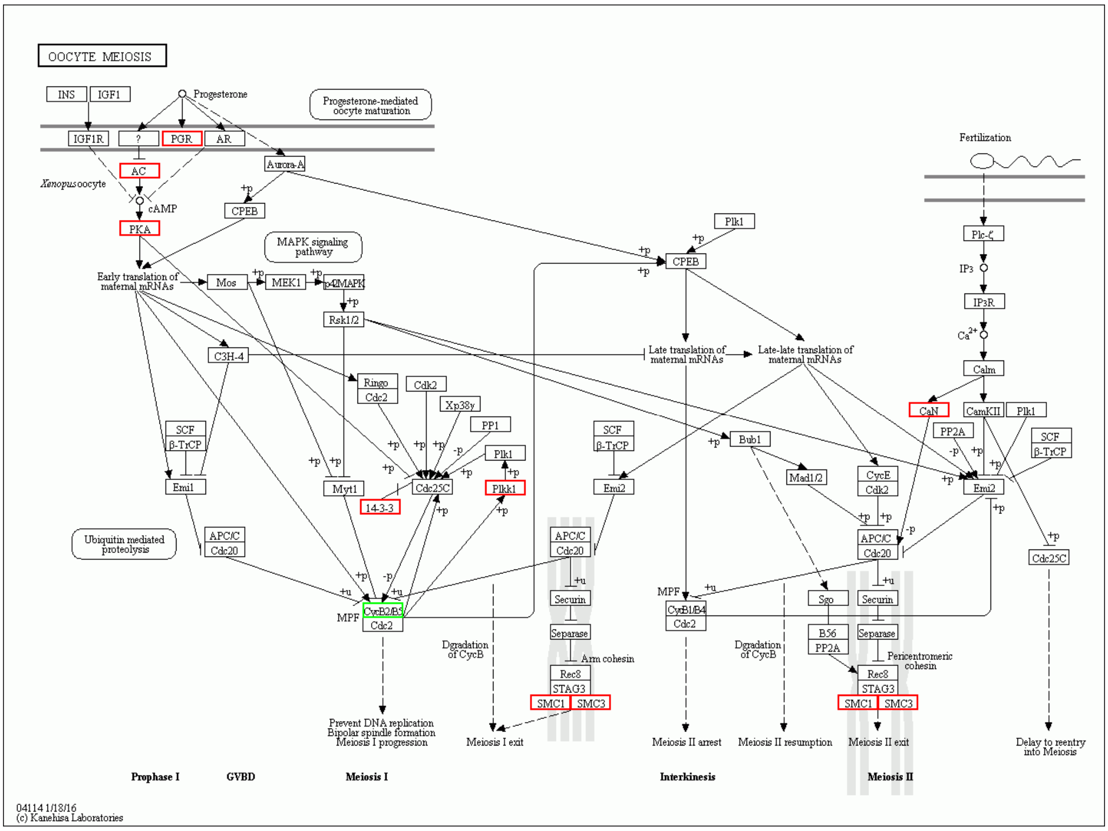

Figure S4. The map of oocyte meiosis pathway in comparison of Control group and Intersex group in RNA-seq.

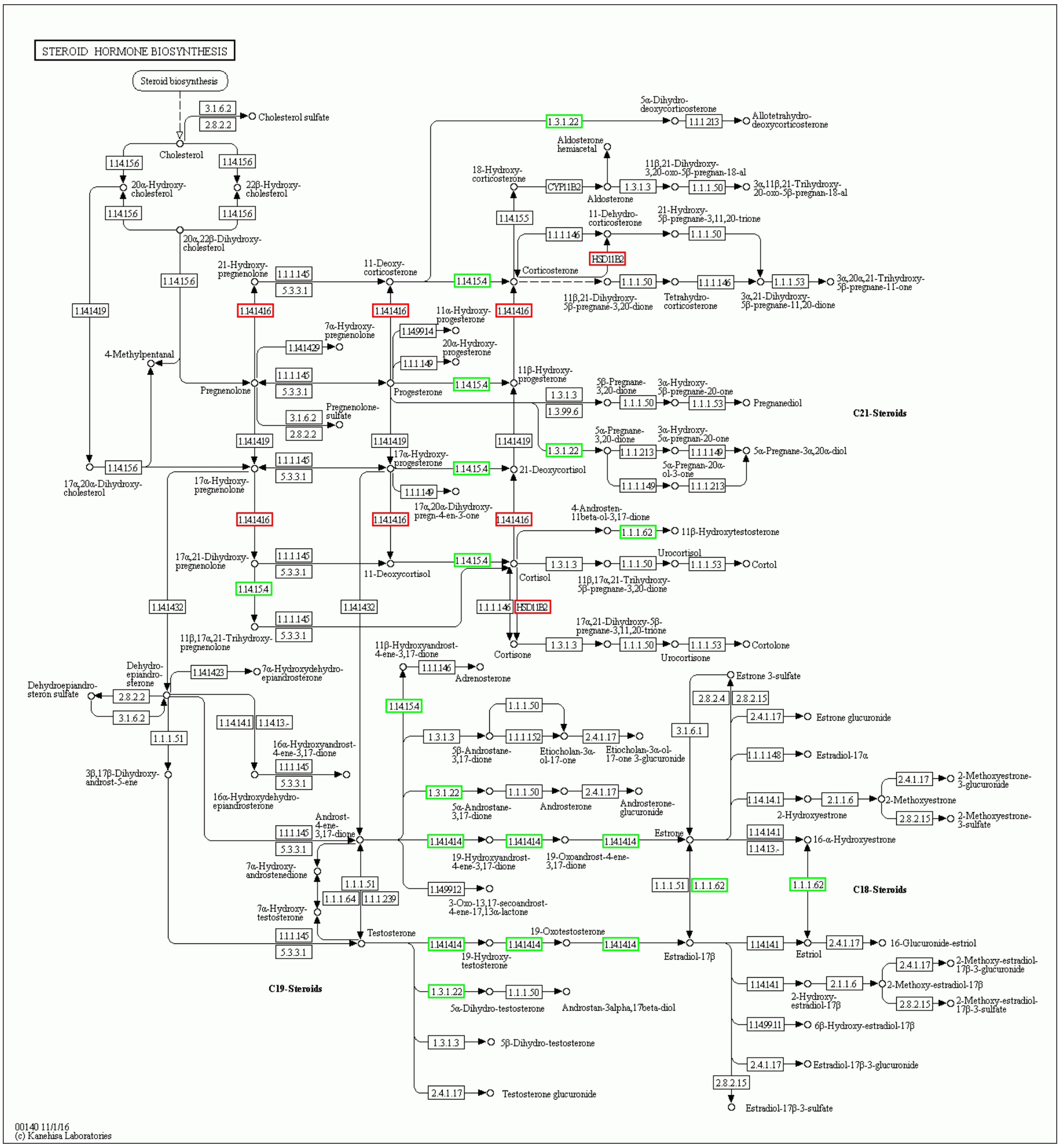

Figure S5. The map of steroid hormone biosynthesis pathway in comparison of Control group and Intersex group in RNA-seq.
